# Paving the Way for the Implementation of a Decision Support System for Antibiotic Prescribing in Primary Care in West Africa: Preimplementation and Co-Design Workshop With Physicians

**DOI:** 10.2196/17940

**Published:** 2020-07-20

**Authors:** Nathan Peiffer-Smadja, Armel Poda, Abdoul-Salam Ouedraogo, Jean-Baptiste Guiard-Schmid, Tristan Delory, Josselin Le Bel, Elisabeth Bouvet, Sylvie Lariven, Pauline Jeanmougin, Raheelah Ahmad, François-Xavier Lescure

**Affiliations:** 1 Infection Antimicrobials Modelling Evolution (IAME), UMR 1137 University of Paris French Institute for Medical Research (INSERM) Paris France; 2 National Institute for Health Research Health Protection Research Unit in Healthcare Associated Infections and Antimicrobial Resistance Imperial College London London United Kingdom; 3 Infectious Diseases Department Bichat-Claude Bernard Hospital Assistance-Publique Hôpitaux de Paris Paris France; 4 Sorbonne Université Paris France; 5 Department of Infectious Diseases University Hospital Souro Sanou Bobo-Dioulasso Burkina Faso; 6 Institut Supérieur des Sciences de la Santé Université Nazi Boni Bobo-Dioulasso Burkina Faso; 7 Service de Bactériologie Virologie University Hospital Souro Sanou Bobo-Dioulasso Burkina Faso; 8 Initiatives Conseil International - Santé Ouagadougou Burkina Faso; 9 Antibioclic Paris France; 10 Institut Pierre Louis d’Épidémiologie et de Santé Publique Sorbonne Université French Institute for Medical Research (INSERM) Paris France; 11 Innovation and Clinical Research Unit Annecy-Genevois Hospital Épagny Metz-Tessy France; 12 Department of General Practice Université Paris Diderot Université de Paris Paris France; 13 School of Health Sciences City, University of London London United Kingdom

**Keywords:** decision support systems, clinical, antibiotic resistance, microbial, drug resistance, microbial, antibiotic stewardship, implementation science, Africa, Western, diffusion of innovation, medical informatics applications

## Abstract

**Background:**

Suboptimal use of antibiotics is a driver of antimicrobial resistance (AMR). Clinical decision support systems (CDSS) can assist prescribers with rapid access to up-to-date information. In low- and middle-income countries (LMIC), the introduction of CDSS for antibiotic prescribing could have a measurable impact. However, interventions to implement them are challenging because of cultural and structural constraints, and their adoption and sustainability in routine clinical care are often limited. Preimplementation research is needed to ensure relevant adaptation and fit within the context of primary care in West Africa.

**Objective:**

This study examined the requirements for a CDSS adapted to the context of primary care in West Africa, to analyze the barriers and facilitators of its implementation and adaptation, and to ensure co-designed solutions for its adaptation and sustainable use.

**Methods:**

We organized a workshop in Burkina Faso in June 2019 with 47 health care professionals representing 9 West African countries and 6 medical specialties. The workshop began with a presentation of Antibioclic, a publicly funded CDSS for antibiotic prescribing in primary care that provides personalized antibiotic recommendations for 37 infectious diseases. Antibioclic is freely available on the web and as a smartphone app (iOS, Android). The presentation was followed by a roundtable discussion and completion of a questionnaire with open-ended questions by participants. Qualitative data were analyzed using thematic analysis.

**Results:**

Most of the participants had access to a smartphone during their clinical consultations (35/47, 74%), but only 49% (23/47) had access to a computer and none used CDSS for antibiotic prescribing. The participants considered that CDSS could have a number of benefits including updating the knowledge of practitioners on antibiotic prescribing, improving clinical care and reducing AMR, encouraging the establishment of national guidelines, and developing surveillance capabilities in primary care. The most frequently mentioned contextual barrier to implementing a CDSS was the potential risk of increasing self-medication in West Africa, where antibiotics can be bought without a prescription. The need for the CDSS to be tailored to the local epidemiology of infectious diseases and AMR was highlighted along with the availability of diagnostic tests and antibiotics using national guidelines where available. Participants endorsed co-design involving all stakeholders, including nurses, midwives, and pharmacists, as central to any introduction of CDSS. A phased approach was suggested by initiating and evaluating CDSS at a pilot site, followed by dissemination using professional networks and social media. The lack of widespread internet access and computers could be circumvented by a mobile app with an offline mode.

**Conclusions:**

Our study provides valuable information for the development and implementation of a CDSS for antibiotic prescribing among primary care prescribers in LMICs and may, in turn, contribute to improving antibiotic use, clinical outcomes and decreasing AMR.

## Introduction

### Antimicrobial Resistance

Antimicrobial resistance (AMR) is a global threat that affects both high-income countries (HIC) and low- and middle-income countries (LMIC). The drivers of AMR, including antibiotic misuse, lack of infection prevention and control, and poor sanitation, particularly affect LMICs [1,2]. Data suggest that 80% to 90% of all antibiotics used in humans are prescribed in primary care [3]. Although antibiotic use has been increasing over the last decade in LMICs [2,4], antibiotic prescribing indicators in primary care for the African region deviate significantly from the World Health Organization’s (WHO) reference targets [5]. Deviations from existing guidelines on antibiotic use, including delays and overuse, are linked both to patients’ behavior and that of health care professionals [6,7]. There is thus a need to find interventions that could improve antibiotic prescribing in primary care to respond to the global threat of AMR.

### Electronic Clinical Decision Support Systems

Electronic clinical decision support systems (CDSS) have been devised to provide prescribers with rapid access to updated information, which is required to make appropriate therapeutic decisions [8]. CDSS can be split into CDSS providing unsolicited information (eg, alerts for drug interactions) and CDSS providing solicited information (eg, diagnostic support systems). Computer representations of patient care guidelines are among commonly used CDSS [9,10]. They allow the prescriber to enter basic clinical information of a patient and access the diagnostic or therapeutic guidelines adapted to the patient’s unique situation. Machine learning CDSS that find their own decision rules from the data are increasingly being developed and may replace knowledge-based CDSS in which human experts define the decision rules in the future [11]. CDSS adapted in primary care have the potential to reduce overall antibiotic use and improve the appropriateness of antibiotic prescribing [12,13]. In LMICs, the introduction of such aids is in the early stages of development [14], but it could have a measurable impact within the context of sparse infectious diseases specialists. Primary care prescribers in LMICs often lack clinical practice guidelines adapted to their setting and do not have access to antimicrobial stewardship programs [15]. Although they may not have access to health care information systems, CDSS providing diagnostic guidance or easy access to therapeutic guidelines may help them make the right decision at the point-of-care [16]. Improving the adequacy of antibiotic prescribing might, in turn, lead to better clinical outcomes and decreased AMR. Moreover, CDSS have the potential to be implemented in multiple health facilities without recruiting new prescribers [17] and could optimize the utilization of resources that are scarce in LMICs.

### Implementation of Clinical Decisions Support System in West Africa

Consequently, the development and implementation of CDSS that fit into clinical work might be part of interventions to mitigate against drivers of AMR in LMICs. However, the adaptation of CDSS for antibiotic prescribing in routine clinical care and their sustainability are often limited [10,18]. As is the case with many innovations in health care, multiple CDSS have been abandoned after their development and initial evaluation because of multiple factors, including the lack of preimplementation work [19]. Indeed, at the global level, the situation analysis for AMR in human health often lack the assessment of technological innovations [20]. Moreover, many CDSS failed to demonstrate a clinical impact because of the low uptake of the CDSS and poor adherence to the generated advice [21,22]. According to the WHO, the adaptation of electronic CDSS is particularly low in LMICs [23]. Interventions to implement CDSS are challenging because of cultural and structural constraints, and these challenges are even more pronounced in primary care where prescribing can be done by a variety of health care professionals or when resources are limited [14,24]. West Africa hosts about 381 million people and includes 16 countries, among them some of the lowest income countries in the world [25,26]. French-speaking West African countries, including Benin, Burkina Faso, Cameroon, Chad, Ivory Coast, Republic of Congo, Guinea, Mali, Niger, Senegal, and Togo, were among the LMICs with the highest increase in global antibiotic consumption between 2000 and 2015 [2]. As highlighted by the WHO, countries in the African region are the least advanced regarding electronic and mobile health [23].

### Objectives

Before developing or implementing a CDSS for antibiotic prescribing in West Africa, we need to understand the contextual, structural, and behavioral determinants that will facilitate or prevent the use of CDSS for antibiotic prescribing. Our study aimed to examine the requirements for a CDSS adapted to the context of primary care in West Africa and to analyze the barriers and facilitators of its implementation and adaptation by prescribers using qualitative methods during a preimplementation workshop.

## Methods

### Study Design

Our aims were (1) to understand the potential benefits and risks of a CDSS for antibiotic prescribing in primary care in West Africa, (2) to analyze the barriers and facilitators of its implementation, and (3) to ensure co-designed solutions for its adaptation and sustainable use. To this end, we organized a workshop with West African antibiotic prescribers that began with a presentation of Antibioclic, a CDSS for antibiotic prescribing currently widely used in France and freely available on the web and as a smartphone app (further details below). We chose to begin the workshop with an example of an existing CDSS rather than with a theoretical description of a CDSS for antibiotic prescribing. This strategy was considered by the organizing team as the most promising to elicit reactions among the participants. The main characteristics of a CDSS were presented, and the organizers went through a number of clinical scenarios on infectious diseases using Antibioclic. Then, the organizers moderated a roundtable discussion with all the participants. During this first-of-its-kind workshop, the participants generated barriers and facilitators to the use of a CDSS such as Antibioclic in West Africa and were asked to hypothesize if its use could lead to benefits or risks, including unexpected events. Participants were specifically asked to debate the challenges in the implementation and adaptation of a CDSS for antibiotic prescribing in West Africa and to co-design solutions.

### Antibioclic—An Example of a Clinical Decision Support System for Antibiotic Prescribing

Antibioclic is a French CDSS for antibiotic prescribing in primary care, [27] targeting 37 common infectious diseases, freely available on the web and as a smartphone app on iOS and Android. It was codeveloped by general practitioners, specialists of infectious diseases, and engineers in 2011. Clinicians can enter the diagnosis of a patient on the website or app, they are then asked a few targeted questions (age group, comorbidities, renal function, breastfeeding, and pregnancy). Finally, they receive a tailored recommendation of antibiotic regimen, dose, and duration according to French national guidelines. Multimedia Appendices 1 and 2 describe how to use Antibioclic respectively with slides or video in English. Antibioclic was developed using a systematic method to transform clinical practice guidelines from the French National Authority for Health and the French Infectious Diseases Society (SPILF) into decision trees in Antibioclic. Antibioclic is updated frequently and modified as soon as a new guideline is published. The number of Antibioclic users in France has steadily increased over the past years from a median (IQR) of 414 (245-494) a day in 2012 to 5365 (2891-5769) a day in 2018, without any saturation to date (Multimedia Appendix 3). The smartphone app has been downloaded 22,970 times on Android and 15,200 times on iOS. More details are described in a recent study [27].

### Workshop Location and Participants

The 3-hour workshop to discuss the implementation and adaptation of a CDSS for antibiotic prescribing in West Africa was organized in Burkina Faso by 2 professors of infectious diseases, 1 from Burkina Faso (AP) and 1 from France (FL). To maximize participation and cost efficiency, the workshop took place during the French-speaking university course *Antibiologie et Antibiothérapie en Afrique Subsaharienne* (*Antibiotic therapy in Sub-Saharan Africa*) organized in Bobo-Dioulasso, Burkina Faso, on June 2019. This course lasts 5 weeks and is organized every year by the University Nazi Boni of Bobo-Dioulasso and the University of Montpellier. The course is open to West African health professionals working on AMR or antibiotic therapy, whether in primary, secondary, or tertiary care, with no selection among participants. Attendance to the workshop was voluntary for the participants of the course.

### Electronic Questionnaire

After the roundtable discussion, an electronic questionnaire with closed- and open-ended questions was given to each participant (Multimedia Appendix 4). The questionnaire allowed the collection of in-depth individual data on the implementation and adaptation of a CDSS for antibiotic prescribing. The questionnaire was codeveloped by a multidisciplinary team including 6 members of the Antibioclic study team: infectious diseases clinicians (AP and FL), a microbiologist (AO), public health specialists (JB and GS) with experience in the West African context, and an implementation science and knowledge mobilization lecturer (RA). The first part collected demographic characteristics and information regarding the use of electronic tools for antibiotic prescribing. The second part displayed open-ended questions about potential outcomes and users of a CDSS for antibiotic prescribing in West Africa and the challenges of its implementation and adaptation.

### Data Analysis

Data from the closed-ended questions of the questionnaire were imported into R software version 3.2.4 (R Foundation for Statistical Computing). Numerical data are presented as absolute numbers, proportions, and median (IQR). The entire roundtable discussion was audio recorded and transcribed verbatim by the AMK France professional company. Data from the roundtable discussion and open-ended questions were analyzed using thematic analysis and were coded to identify key categories, which were developed into themes using NVivo 12 software (QSR International). The analysis of questionnaires and the roundtable discussion supported cross-validation and triangulation of the findings.

### Ethical Considerations

Written consent was obtained from each participant. No distinguishable personal information was recorded, and all the data were analyzed anonymously. This survey was approved by the research ethics committee of Centre Muraz (Health Research Institute, Bobo-Dioulasso, Burkina Faso) and was compliant with the European General Data Protection Regulation.

## Results

### Participants

All 47 participants of the course, 19 women and 38 men with a median (IQR) age of 31 (30-38) years, participated in the workshop and completed the questionnaire (Table 1). Most of the participants (35/47, 74%) were from Burkina Faso, but 8 other West African countries were represented. Approximately half of the participants (24/47, 51%) worked in a university hospital, whereas the others worked in general hospitals (14/47, 30%), public health institutes (4/47, 9%), dispensary (1/47, 2%), private hospital (1/47, 2%), or pharmacies (1/47, 2%). The low number of participants working as primary care prescribers may be related to a more difficult access to university degrees. The specialty of the participants was general practice (21/47, 45%), microbiology (13/47, 28%), pharmacy (4/47, 9%), anesthesiology and intensive care (3/47, 6%), infectious diseases (2/47, 4%), and neurosurgery (2/47, 4%). Only 26% (12/47) of participants, all in Burkina Faso, stated that they used national guidelines to prescribe antibiotics. The others used French guidelines (20/47, 43%), WHO guidelines (20/47, 43%), or local hospital guidelines (4/47, 9%).

**Table 1:** Demographic characteristics of participants (N=47).

Characteristics			Values	
Age (years), median (IQR)			31 (30-38)	
**Gender, n (%)**				
		Men		28 (60)
		Women		19 (40)
**Country of practice, n (%)**				
	Burkina Faso			35 (74)
	Togo			3 (6)
	Senegal			2 (4)
	Mali			2 (4)
	Gabon^a^			1 (2)
	Guinea			1 (2)
	Guinea-Bissau			1 (2)
	Ivory Coast			1 (2)
	Niger			1 (2)
**Working structure, n (%)**				
	University hospital			24 (51)
	General hospital			14 (30)
	Public health institute			4 (9)
	Dispensary			1 (2)
	Private hospital			1 (2)
	Pharmacy			1 (2)
**Medical specialty, n (%)**				
	General practice			21 (45)
	Microbiology			13 (28)
	Pharmacist			4 (9)
	Anesthesiology and intensive care			3 (6)
	Infectious diseases			2 (4)
	Neurosurgery			2 (4)
**Guidelines used for clinical practice, n (%)**				
	French			20 (43)
	World Health Organization			20 (43)
	National^b^			12 (26)
	Hospital			4 (9)
	American			2 (4)
	European			2 (4)
	Portuguese			1 (2)

^a^Central Africa.

^b^The 12 participants were from Burkina Faso.

### Current Use of Technology and Clinical Decision Support Systems

**Figure 1:**
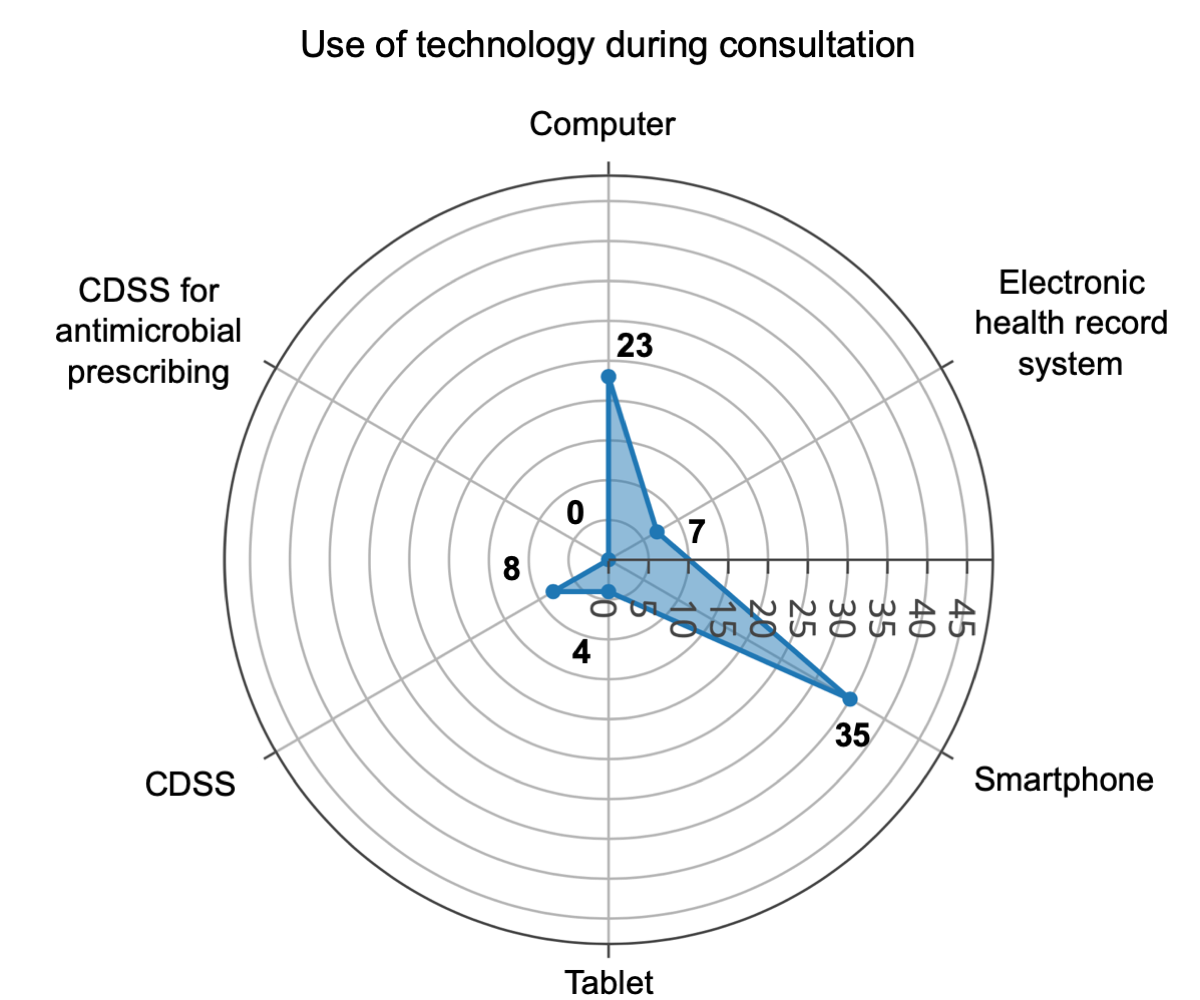
Current use of technology during consultation among participants. CDSS: clinical decision support systems.

Most of the participants (35/47, 74%) had a smartphone with internet access during their clinical consultations, but less than half (23/47, 49%) of the participants had access to a computer, and 15% (7/47) of the participants had no access to any electronic tools during their consultations. Most of the clinicians did not use an electronic health record system (39/47, 83%) or CDSS (36/47, 77%) for any clinical decision making during their clinical consultations. The only CDSS mentioned by the participants was a tool for basic medical calculations and a tool that displays medical cards. No CDSS for antibiotic prescribing (nor for any antimicrobial drug) was used by the participants (Figure 1).

### Potential of a Clinical Decision Support Systems for Antibiotic Prescribing

**Figure 2:**
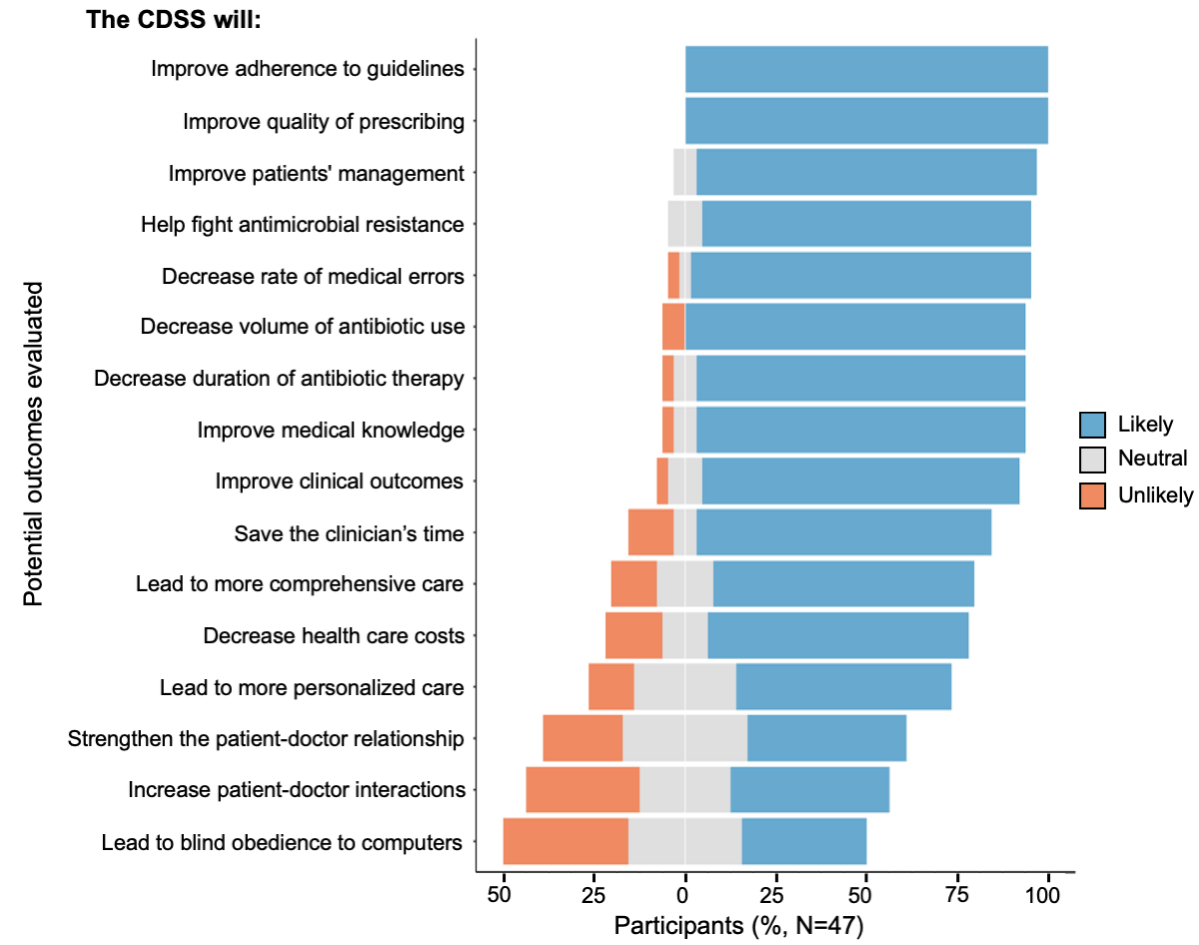
Expected outcomes of a clinical decision support system for antibiotic prescribing. CDSS: clinical decision support systems.

Most of the participants expected favorable outcomes from the implementation of a CDSS for antibiotic prescribing in primary care in their countries (Figure 2). They thought a CDSS was likely to improve the quality of antibiotic prescribing (47/47, 100%), improve adherence to guidelines (47/47, 100%), improve the management and care of patients (44/47, 94%), decrease the number of medical errors (44/47, 94%), improve the volume of antibiotic use (44/47, 94%), improve the duration of antibiotic therapy (43/47, 91%), fight against AMR (43/47, 91%), increase the medical knowledge of users (43/47, 91%), and save time during consultation (38/47, 81%). Participants disagreed on the fact that such a tool could strengthen the physician-patient relationship (20/47, 43% judged it likely; 11/47, 23% unlikely; and 16/47, 34% were neutral) or could lead to blind obedience to electronic tools from prescribers (16/47, 34% judged it likely; 16/47, 34% unlikely; and 15/47, 32% were neutral).

During the roundtable discussion and through the open-ended questions, the participants identified a number of consequences of a CDSS on a wider scale. Participants underlined the lack of antimicrobial stewardship teams in West Africa. They hypothesized that a CDSS could help many isolated structures to have access to antibiotic prescribing recommendations and could relieve existing antimicrobial stewardship teams of simple antibiotic prescribing advice and let them focus on complex cases. One participant noted that a CDSS could be used to increase surveillance capability in primary care. Indeed, its use could be monitored and lead to reports on the incidence of infectious diseases and the rate of antibiotic prescriptions. The participants agreed that this is particularly important in West African countries where epidemiological data are scarce. The use of French guidelines was not seen as a barrier to a CDSS. Indeed, most of the participants declared that they did not have national guidelines to prescribe antibiotics in their country. However, the participants pointed out that a successful CDSS could have a positive impact in encouraging national societies to develop guidelines that could then be integrated into the CDSS. More broadly, they agreed that this CDSS could help to increase the awareness of health professionals on the importance of appropriate prescribing and the risks of AMR. The participants also agreed that a CDSS could be used in the initial training and continuous medical education of health professionals by updating antibiotic prescribing knowledge:

If you use it continually, you certainly update your knowledge.

General practitioner in Burkina Faso

Regarding the scope of a CDSS for antibiotic prescribing, the participants stated that it could have a positive impact on the management of urinary tract infections (84%); skin and soft tissue infections (84%); gastrointestinal tract infections (83%); genital infections (80%); pneumonia (77%); meningitis (70%); ear, nose, and throat infections (70%); dental infections (67%); and surgical site infections (61%). The participants identified a wide diversity of potential users of a CDSS for antibiotic prescribing in West Africa, including general practitioners, infectious diseases specialists, physicians in other specialties, pharmacists, nurses, clinical microbiologists, and midwives.

### Challenges

The participants identified a number of challenges and proposed a number of solutions for the adaptation of a CDSS for antibiotic prescribing in a West African setting (Table 2), and these are grouped by system level.

**Table 2:** Challenges and potential solutions for the development and implementation of a clinical decision support system in the West African context.

Level	Challenges to CDSS^a^ development	Challenges to CDSS implementation	Potential solutions
Country level	Scarce epidemiological data on the prevalence and incidence of infectious diseases and the level of antimicrobial resistance	N/A^b^	Encouraging studies to better analyze the local and regional epidemiology Developing and updating the CDSS according to local and regional epidemiology regarding infectious diseases, microbiology, and antimicrobial resistance Including tuberculosis and common parasitic diseases
	Lack of national guidelines	N/A	The CDSS should follow local, regional, and national guidelines where they exist. If they do not, the CDSS could follow French or WHO^c^ guidelines as they are used by most participants The CDSS should be developed for the subcontinent of West Africa and then could be further adapted to each country To easily adapt the CDSS to local and national guidelines, the programming and code of the CDSS should be in open access
	Limited availability of diagnostic tests and antibiotics	N/A	Adapting the suggestions to locally available diagnostic tests and antibiotics by working with national scientific societies
Health care structure level	N/A	Lack of internet access and information technology infrastructure	Development of an offline mode of the CDSS Development of a mobile version on iOS and Android Increasing the availability of computers and internet access in West Africa
	N/A	Independently operating and geographically isolated health structures such as dispensaries	Pilot testing of the CDSS in a primary care structure linked to an academic hospital before disseminating the tool to other health structures Field communication with primary care prescribers Using the network of the Ministry of Health
Individual level: clinicians and patients	Diversity of training needs for primary care prescribers	N/A	Co-designing the CDSS with general practitioners, nurses, midwives, microbiologists, dentists, and pharmacists Allowing for different modules of access for health professionals to meet the different information needs
	N/A	Lack of awareness and training about CDSS	Dedicated training for primary care prescribers Communicating through scientific and professional societies, using traditional and social media Involving all the stakeholders, including health authorities, the Ministry of Health, and the media
	N/A	Risk of increasing self-treatment with antibiotics as they are available without prescription in most West African countries	Limiting access to registered health professionals (disagreement between participants) Regulating access to antibiotics without prescription
	N/A	Risk of deskilling and dependency of prescribers	Improving the training of prescribers about antibiotic prescribing
	N/A	Risk to lose patients’ confidence by following the advice of an electronic tool	Education of patients about the need to use reference books or electronic sources to provide the best care Ensuring the independence of the tool from pharmaceutical companies

^a^CDSS: clinical decisions support system.

^b^N/A: not applicable.

^c^WHO: World Health Organization.

#### Country Level

At the country level, the participants pointed out the scarcity of epidemiological data in West African countries, the difference between epidemiology of diseases in HICs and LMICs, the lack of national guidelines, and the limited availability of diagnostic tests and antibiotics. To produce computer-interpretable clinical guidelines, CDSS developers need to have access to clinical practice guidelines. For countries where national guidelines for infectious diseases management do not exist, the participants first suggested the development of a CDSS based on French or WHO guidelines to encourage the establishment of national guidelines. To address the specific needs of primary care prescribers in West Africa, the CDSS should not only target the management of common bacterial diseases but also the management of parasitic diseases and tuberculosis. Participants stressed the importance of tailoring the CDSS to local capacity and infrastructure, such as resource availability and antibiotics, with the help of national scientific societies.

#### Health Care Structure Level

The first challenge at the organization level was the lack of internet access and information technology (IT) infrastructure. Taking into account the wide availability of smartphones with internet access, the participants highlighted major opportunities through the development of mobile phone versions of CDSS (on iOS and Android) with an offline mode. The second challenge was the important number of independently operating and geographically isolated health structures, such as dispensaries, in the West African health system. To reach these structures, participants suggested the involvement of relevant stakeholders, including health authorities, the Ministry of Health, scientific societies, the media, and opinion leaders, using traditional and social media:

It is important to use the traditional networks of the Ministry of Health where information goes down hierarchically but also social media that are more and more used.

Physician in a university hospital, Burkina Faso

The organization of a phased approach was suggested by initiating and evaluating CDSS in a pilot primary care structure linked to an academic hospital followed by dissemination to other health structures:

You have to show people that it's used by people like them starting with a pilot test site.

Physician in a general hospital, Burkina Faso

#### Individual Level: Clinicians and Patients

The diversity of primary care prescribers who have different levels of decision support needs was identified as a challenge to developing effective CDSS. A physician working in a general hospital stated that in West Africa “nurses see a lot of patients, certainly more than physicians” highlighting the need to codevelop the CDSS with primary care health care professionals who frequently prescribe antibiotics, such as general practitioners, nurses, midwives, microbiologists, dentists, and pharmacists. The implementation of a CDSS in West Africa should take into account the lack of widespread knowledge on the use of CDSS by organizing training sessions with primary care prescribers. One participant said that some health care professionals or patients could be refractory to new technologies, but this was not considered a major barrier by the other participants. The participants identified a number of facilitators for the adaptation of a CDSS by primary care prescribers and patients, including the independence of the tool from pharmaceutical companies, codevelopment with users, free access to the CDSS, and good usability.

The most discussed unexpected consequence was that an open access CDSS such as Antibioclic could amplify self-medication of antibiotics due to availability without prescription:

My point of view is really mixed. This represents an undeniable progress for antibiotic prescribing but in the context of our country, there is a non-negligible risk of self-medication by non-healthcare professionals.

General practitioner, Burkina Faso

A very good tool for the treatment of infections but which could be dangerous in the southern countries because of self-medication and the accessibility of antibiotics without prescription.

Physician in a university hospital, Burkina Faso

In this context, the CDSS may be used as a substitute for expert health care opinion. However, some participants highlighted the fact that much information is already available on the web and that Antibioclic is already available, including in Burkina Faso, with no reported use by patients. In fact, there was uncertainty regarding if implementing a CDSS in West Africa would amplify the phenomenon of self-medication or would help by providing a reliable source to patients who would want to self-medicate anyway:

Some patients will buy antibiotics without prescription, with or without the tool.

General practitioner, Burkina Faso

On the contrary, if patients have access to national recommendations, this reinforces and this even facilitates your message.

Professor in a university hospital, Burkina Faso

A consensus emerged that the best solution is to increase efforts to limit access to antibiotics without prescription.

## Discussion

### Principal Findings

**Figure 3:**
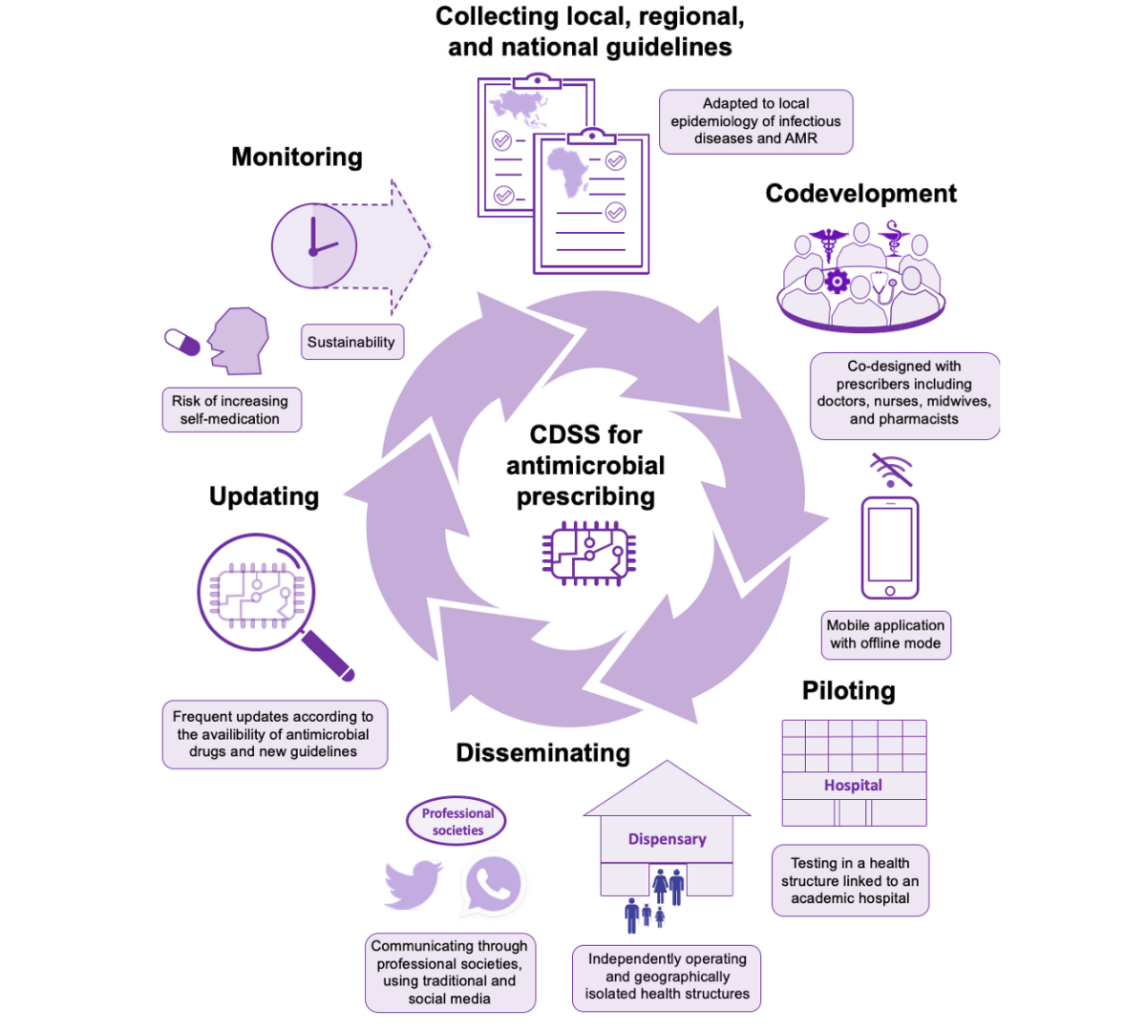
Key steps for the development and implementation of a clinical decision support systems for antibiotic prescription in low- and middle-income countries. AMR: antimicrobial resistance; CDSS: clinical decision support systems.

We conducted a preimplementation workshop with West African health professionals to examine the requirements for a CDSS adapted to the context of primary care in their countries. The use of CDSS for antibiotic prescribing (and other clinical decisions, in fact) is currently missing, but the importance and need for such tools to support antibiotic prescribing in primary care were stressed by all participants. Regarding the implementation of a CDSS in primary care, the participants encouraged a procedure of co-designing the tool with health professionals and stakeholders involved in antibiotic prescribing and initiation via a primary care pilot site, which is linked to an academic hospital (Figure 3). The diffusion of the CDSS would be more successful if supported by networks of medical specialists and the Ministry of Health but should also take advantage of social media because of its high use among health care professionals. The lack of widespread internet access and computers during consultation was a barrier to the use of the CDSS, but it could be circumvented by the development of a mobile app with an offline mode. The CDSS should be tailored to the local epidemiology of infectious diseases and AMR, and the high prevalence of malaria, tuberculosis, and HIV in West Africa should be taken into account when developing the CDSS [28]. The availability of diagnostic tests and antibiotics may differ between West African countries and may vary over time, requiring frequent updates of the CDSS.

The participants pointed out the potential risk of self-medication in countries in which antibiotics can be bought with no prescription. Such a risk should certainly be anticipated and monitored, but most of the participants did not think that the CDSS should have restricted access. On the contrary, some participants thought that a CDSS could provide an opportunity to strengthen the education of the general public about antibiotic prescribing and AMR. Development and implementation of a CDSS was seen to be associated with a number of benefits related to improving clinical care and reducing AMR and also to strengthening and updating practitioners’ knowledge of antibiotic prescribing recommendations, encouraging the development of national guidelines, and developing surveillance capabilities in primary care. Indeed, the implementation of a CDSS for antibiotic prescribing could be coupled with measures that allow an increase in the capability of surveillance of antibiotic use. Examples include real-time monitoring of requests expressed on the platform and extracting reports about antibiotic use [27,29]. Moreover, the implementation of a CDSS in the community could have implications beyond antibiotic prescribing in primary care. Indeed, primary care is the gateway to secondary and tertiary care, and AMR in primary care circulates in the whole health system [30].

### Comparison With Prior Work

The lack of preimplementation and co-design work concerning the assessment of CDSS for antibiotic prescribing has been reported in recent reviews [10,31]. More broadly, research on the implementation strategies for CDSS in primary care is lacking [32]. A qualitative design involving 33 face-to-face interviews with general practitioners has been used to codevelop computer-delivered interventions to promote guidelines for antibiotic prescribing in the United Kingdom. The authors focused on the technical development of prompts and the determinants of their acceptability but did not study the implementation of the CDSS in clinical practice [33]. Postimplementation research found that the key factor for the use of this CDSS by general practitioners in primary care was their awareness of the implementation of the system within their practice [22]. The lack of end-user engagement with implementation and the rigidity of the guidelines incorporated in the prompts were barriers to the effective adaptation of the system, stressing the importance of a systematic and theoretically informed process of implementation for CDSS [34]. The nonadaptation, abandonment, scale-up, spread, and sustainability framework has been developed to support the implementation of technological innovations in health and social care [19]. It lists challenges in 7 domains: the condition or illness, the technology, the value proposition, the adopter system (comprising professional staff, patient, and lay caregivers), the organization(s), the wider (institutional and societal) context, and the interaction and mutual adaptation between all these domains over time.

Few studies have focused on CDSS for the management of infectious diseases in LMICs and in particular in West Africa. A recent review identified 6 CDSS for the management of febrile children in primary care in LMICs [35]. In a pilot cluster-randomized controlled study, the clinical algorithm for management of childhood illness available on smartphones (e-ALMANACH) increased the number of children screened for red flags and decreased antibiotic prescription in Tanzania [36]. This result was confirmed in another controlled study in which the use of e-ALMANACH to manage children improved clinical outcomes and reduced antibiotic prescription by 80% [37]. Electronic point-of-care tool, an electronic algorithm using point-of-care testing results including C-reactive protein and procalcitonin, was shown to improve the clinical outcomes of children with febrile illnesses as compared with the e-ALMANACH CDSS [38,39]. Another study described a protocol for the implementation on a large scale in Burkina Faso of an electronic tool for integrated management of childhood illnesses [40]. The results of this trial have not been published yet. In general, few data are available on the adaptation and sustainability of such interventions. One team analyzed the implementation cost of a CDSS for antenatal and delivery care and found that most of the cost resulted from the recruitment and training of nurses and midwives using the CDSS and from buying laptops [41]. A CDSS that is easily used by health care professionals and is available as a free mobile app could lead to a decreased implementation cost. A study in Burkina Faso confirmed a positive attitude toward electronic decision support systems and the need for simplicity and good usability of the tool. They found similar barriers, such as the lack of infrastructure and IT systems and the limited availability of drugs and diagnostic tests [42]. The development of CDSS in LMICs could be facilitated by the development of mobile health [43] and by sharing open-source codes [44]; however, other challenges remain: countries often have to buy a back-office or another hosting solution and have to pay for the maintenance of the server [45,46]. These costs, if not anticipated, can limit the sustainability of CDSS.

### Limitations

The participants stressed the diversity of prescribers and health structures in West Africa that must be taken into account when codeveloping the CDSS. However, most of the participants in our workshop were physicians specialized in general practice working in Burkina Faso university hospitals. This bias was related to our recruitment of participants via a university degree organized by French and Burkina Faso universities. We, thus, need to involve more primary care prescribers such as nurse prescribers, midwives, and physicians working in dispensaries to allow for a broader view of primary care antibiotic prescribing. Indeed, prescribing habits may vary between professional groups [47,48], and we would like to capture potential differences among prescribers concerning the use, needs, and expectations regarding CDSS for antibiotic prescribing.

To open the discussion, we decided to provide an example of a currently used CDSS. Indeed, as we anticipated that most of the participants had no experience using electronic decision support systems for antibiotic prescribing, we wanted to show them an existing CDSS and collect opinions. This approach was likely to influence the answers of participants and the discussion during the roundtable discussions. However, we think that the barriers and facilitators identified in the study are not limited to Antibioclic. Indeed, the contextual barriers that have been found have been described in other studies. Nonprescription dispensing of antibiotics in the community has been widely described both in LMICs [49,50] and in HICs [51]. In a recent review, the authors did not find any clinical practice guideline applicable to prehospital care in LMICs among 276 guidelines [15]. For countries where national guidelines for the management of infectious diseases do not exist, the participants suggested the development of a CDSS first based on French or WHO guidelines to encourage the establishment of national guidelines.

The governance system and public health priorities could be important additional factors that need to be studied in West Africa before the implementation of a CDSS. Work has been done in European countries about governance to address AMR [52], and relevant frameworks for assessing this important factor exist [20,53]. However, the analysis and governance frameworks for antibiotic prescribing often lack the assessment of technological innovation, such as electronic decision support. Including CDSS in the national action plan as in the United Kingdom’s 2019-2024 national plan that targets to “be able to report on the percentage of prescriptions supported by a diagnostic test or decision support tool by 2024” is probably an important step toward the development and implementation of support systems [54]. We are not aware of Western African national action plans mentioning the development and use of CDSS.

### Conclusions

Preimplementation research is needed to ensure that CDSS are adapted to the context in which they are deployed. Our study provided valuable information to develop and implement a CDSS for antibiotic prescribing among primary care prescribers in LMICs. We plan to organize workshops to co-design a CDSS tailored to the context of primary care in West Africa. Optimizing the use of antibiotics in primary care may have beneficial consequences for the entire health system and can contribute to limiting the spread of AMR. Most of the barriers and facilitators that we identified may easily relate to a broad spectrum of CDSS, including systems for clinical decisions other than antibiotic prescribing.

## Appendix

Multimedia Appendix 1 How to use Antibioclic (slides).

Multimedia Appendix 2 How to use Antibioclic (video).

Multimedia Appendix 3 Antibioclic users.

Multimedia Appendix 4 Electronic questionnaire.

## Abbreviations

AMR: antimicrobial resistance
CDSS: clinical decisions support system
HIC: high-income countries
LMIC: low- and middle-income countries
SPILF: French Infectious Diseases Society
WHO: World Health Organization
