# Supplementary material for: Paving the Way for the Implementation of a Decision Support System for Antibiotic Prescribing in Primary Care in West Africa: Preimplementation and Co-Design Workshop With Physicians
Source: J Med Internet Res. 2020 Jul 20;22(7):e17940. doi: 10.2196/17940 (PMC7400049; doi:10.2196/17940)
Supplement: Multimedia Appendix 1 [file jmir_v22i7e17940_app1.pptx]

## Slide 1
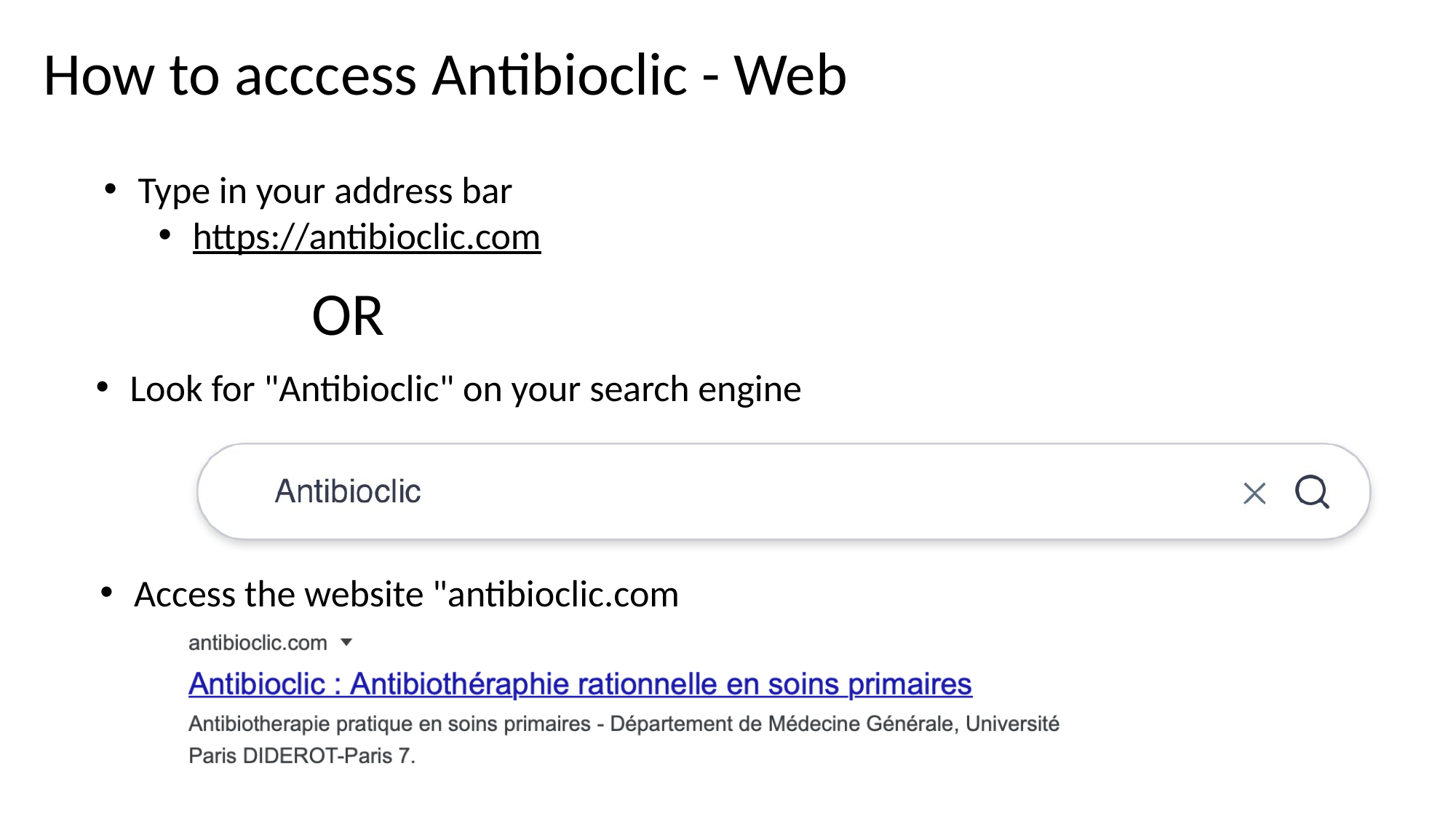

How to acccess Antibioclic - Web
Type in your address bar
https://antibioclic.com
OR
Look for "Antibioclic" on your search engine
Access the website "antibioclic.com

## Slide 2
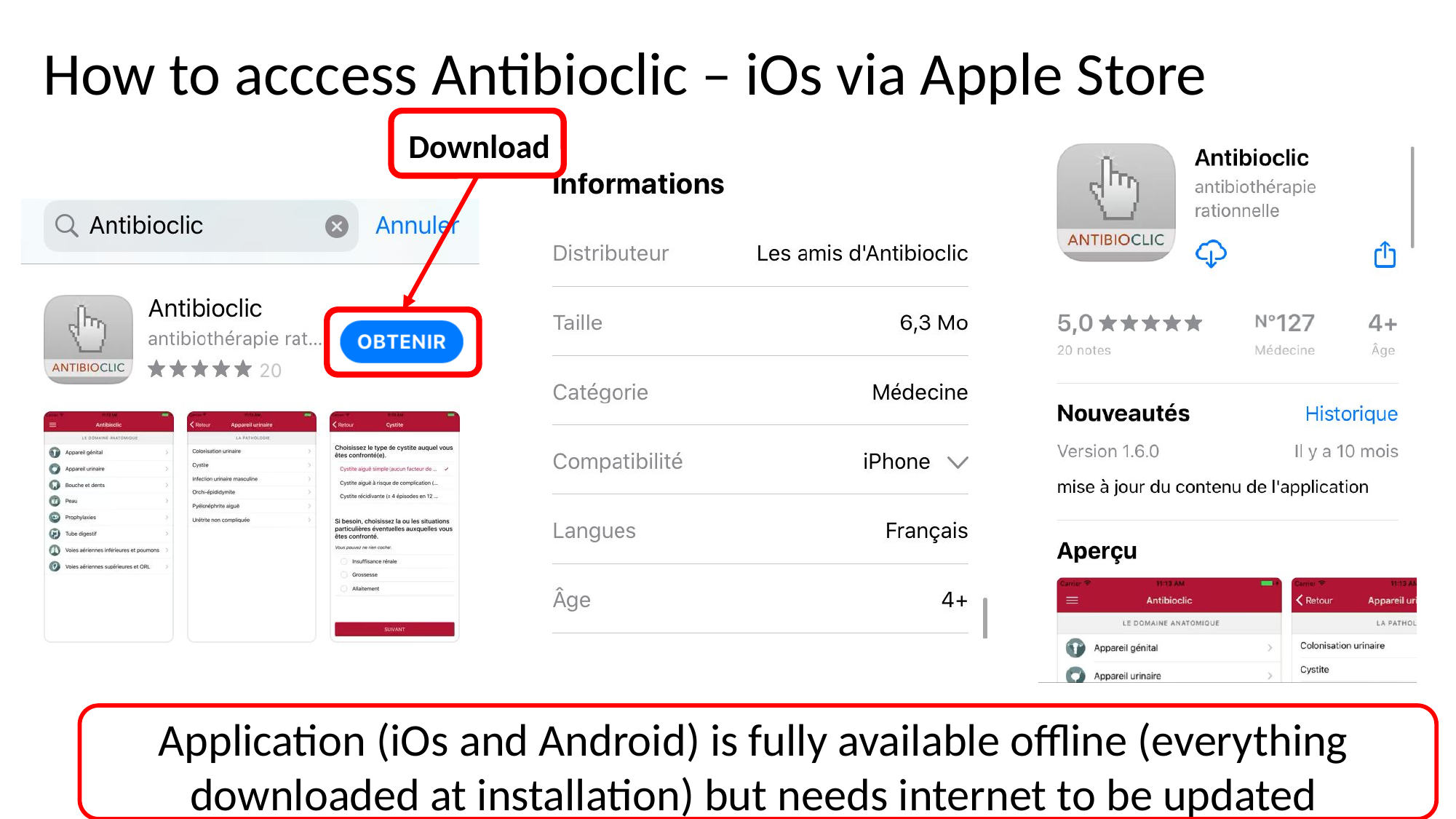

How to acccess Antibioclic – iOs via Apple Store
Download
Application (iOs and Android) is fully available offline (everything downloaded at installation) but needs internet to be updated

## Slide 3
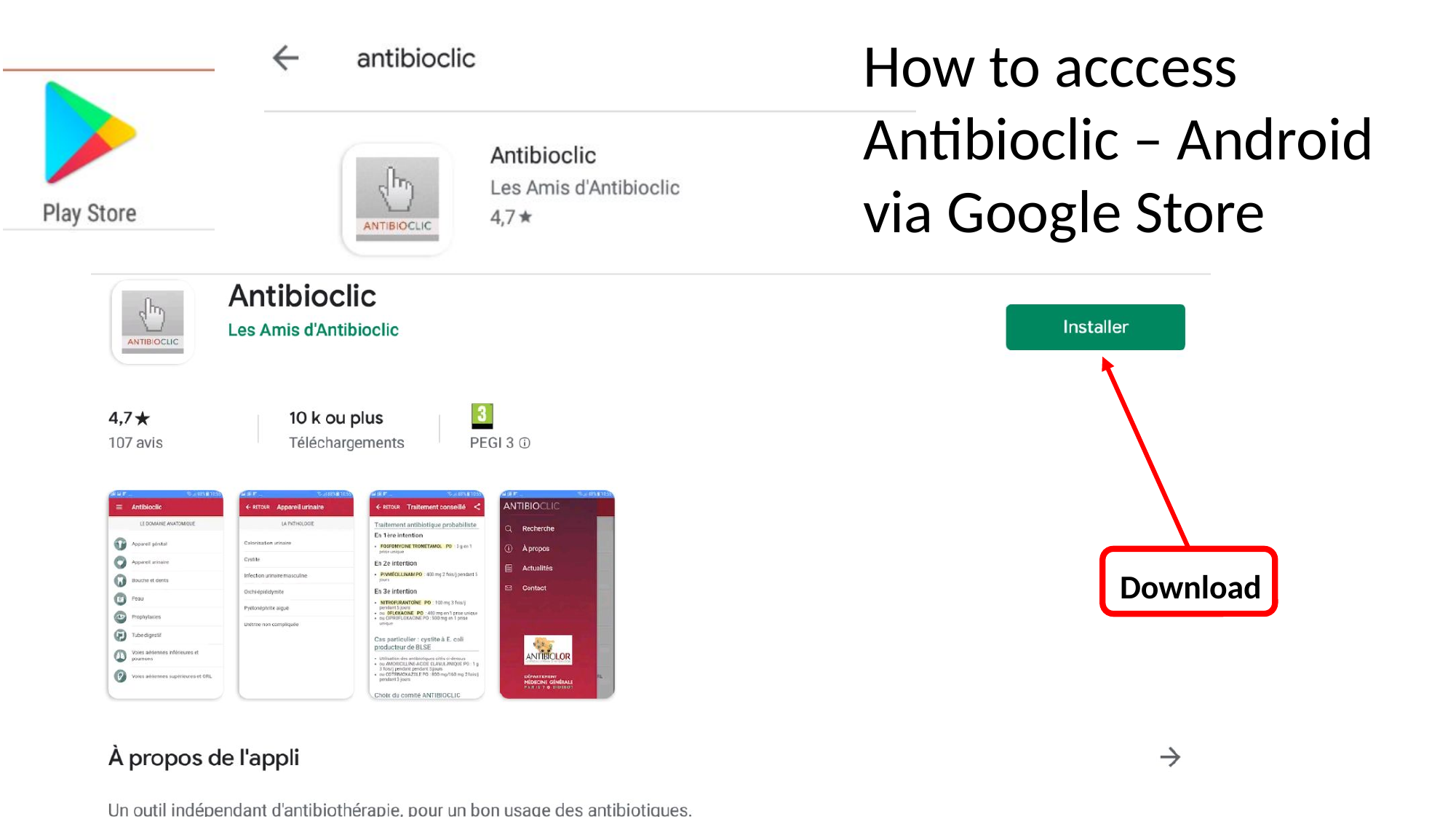

How to acccess Antibioclic – Android via Google Store
Download

## Slide 4
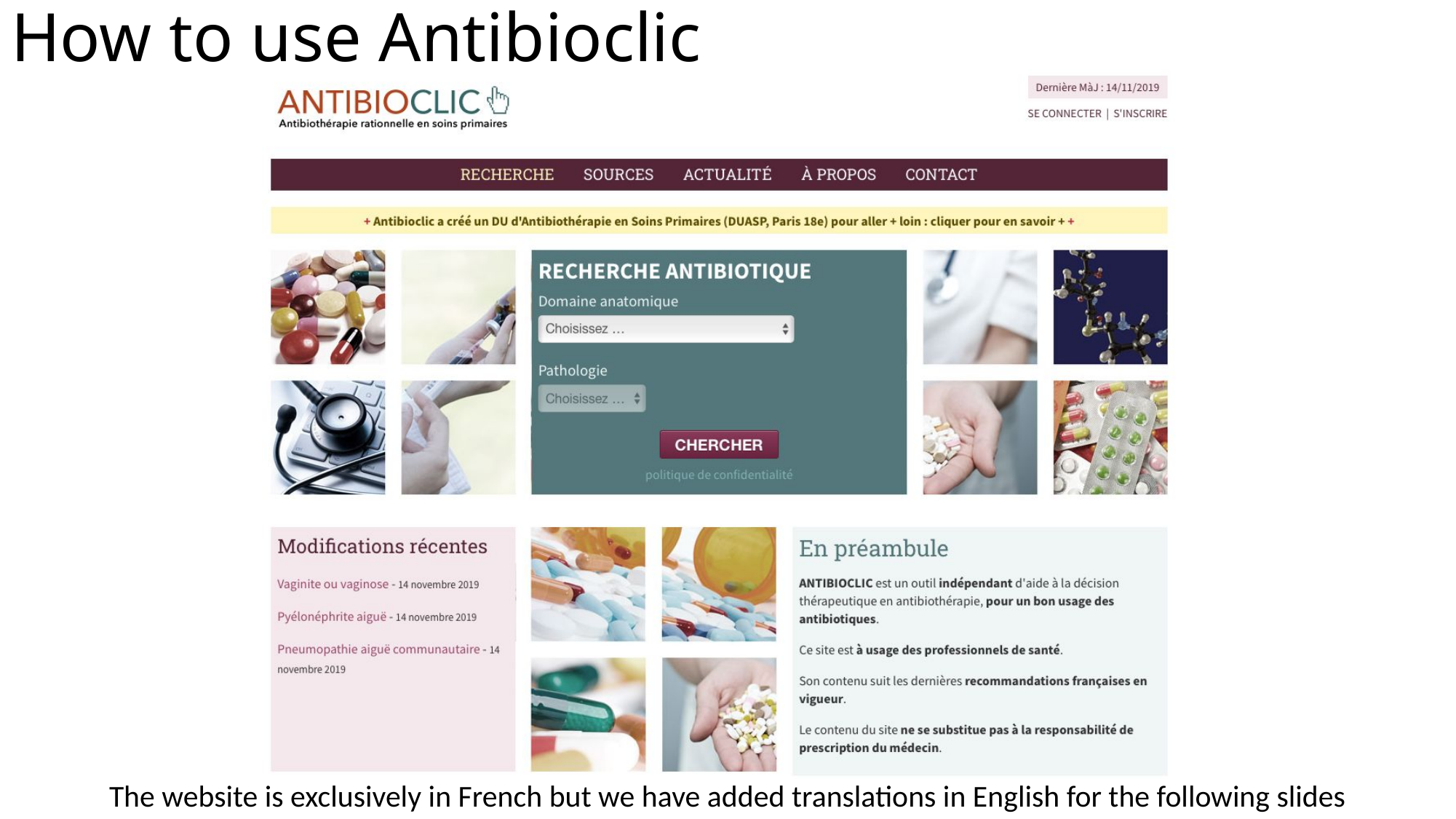

# How to use Antibioclic
The website is exclusively in French but we have added translations in English for the following slides

## Slide 5
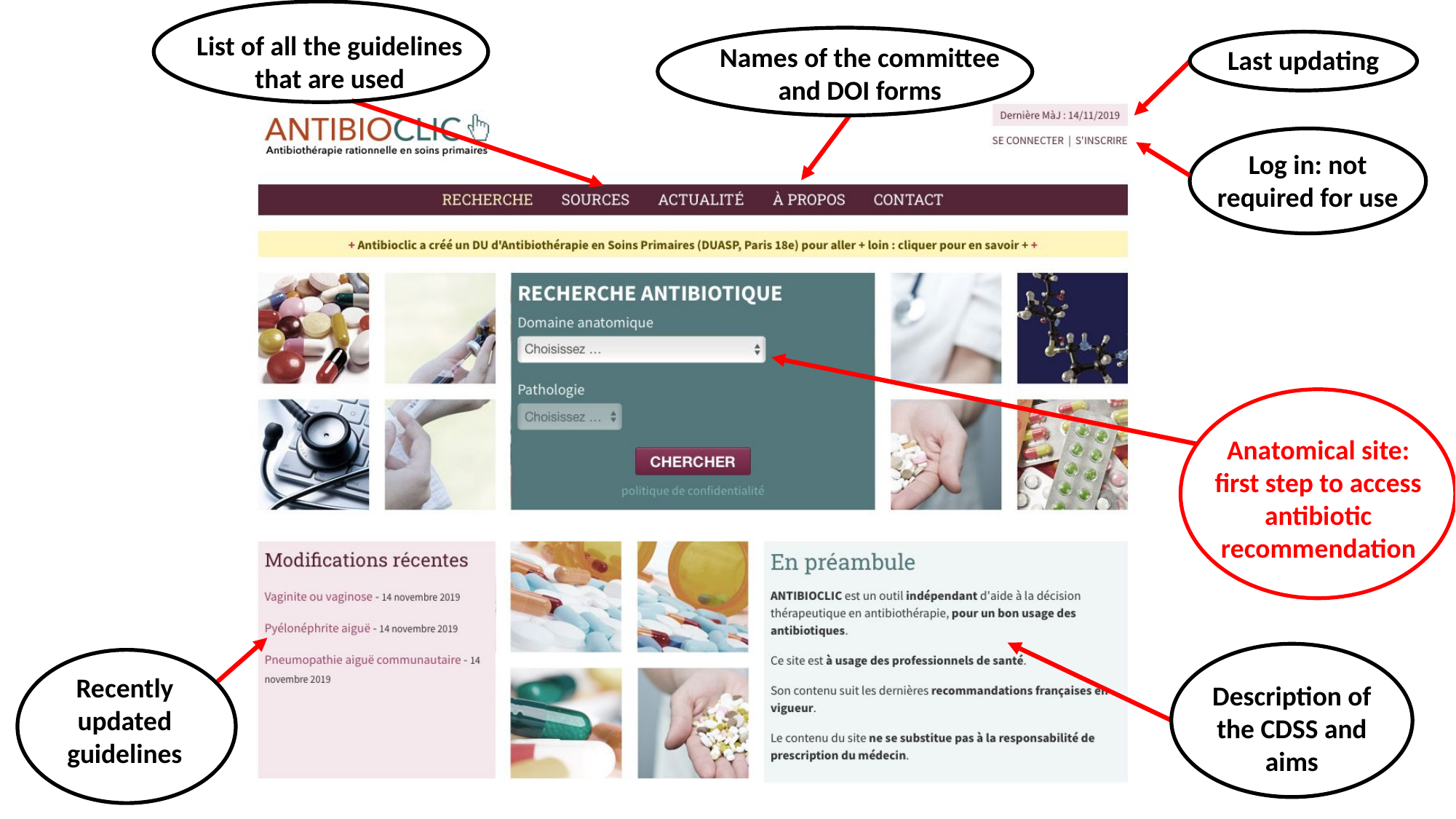

List of all the guidelines that are used
Names of the committee and DOI forms
Last updating
Log in: not required for use
Anatomical site: first step to access antibiotic recommendation
Recently updated guidelines
Description of the CDSS and aims

## Slide 6
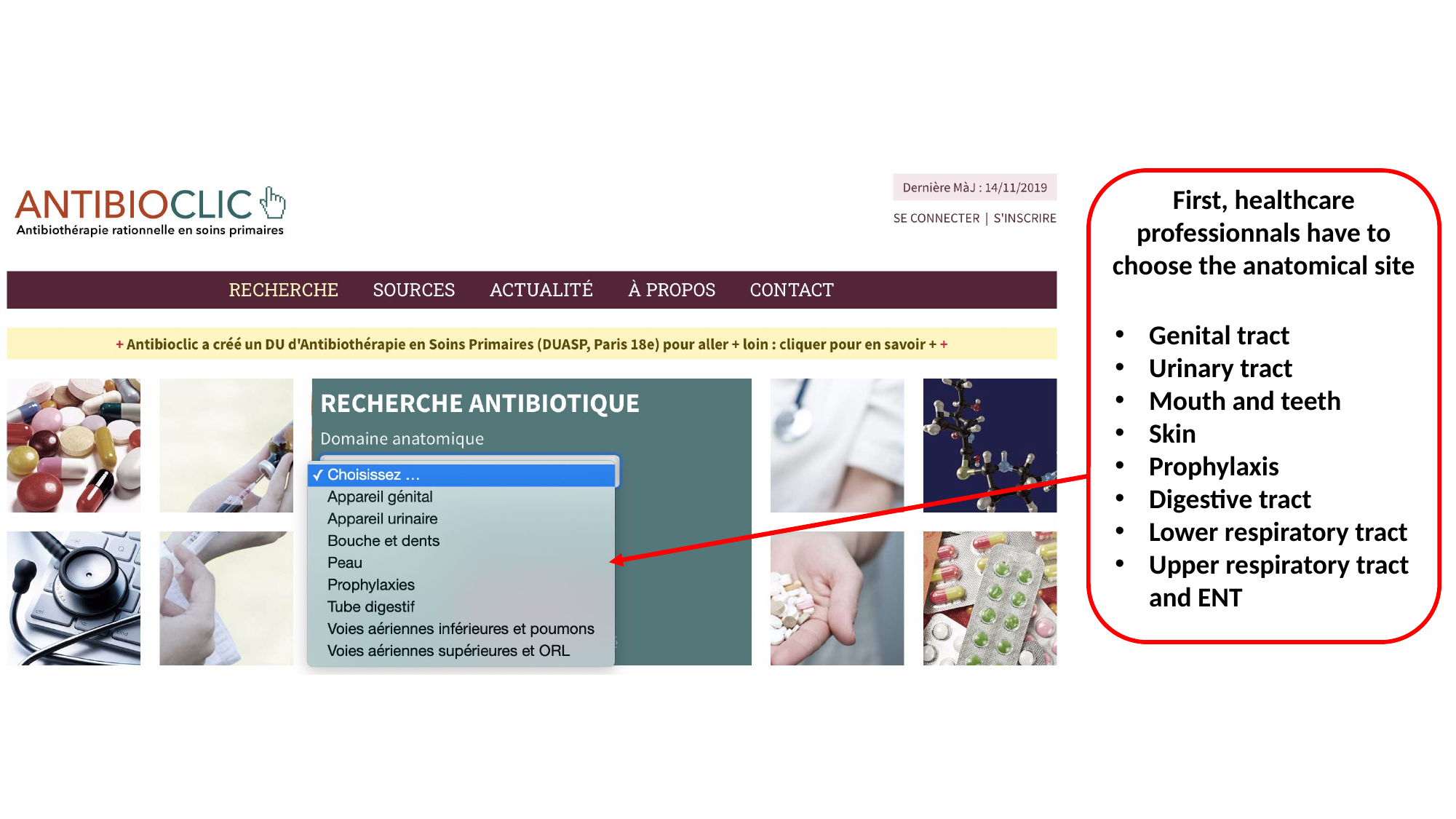

First, healthcare professionnals have to choose the anatomical site
Genital tract
Urinary tract
Mouth and teeth
Skin
Prophylaxis
Digestive tract
Lower respiratory tract
Upper respiratory tract and ENT

## Slide 7
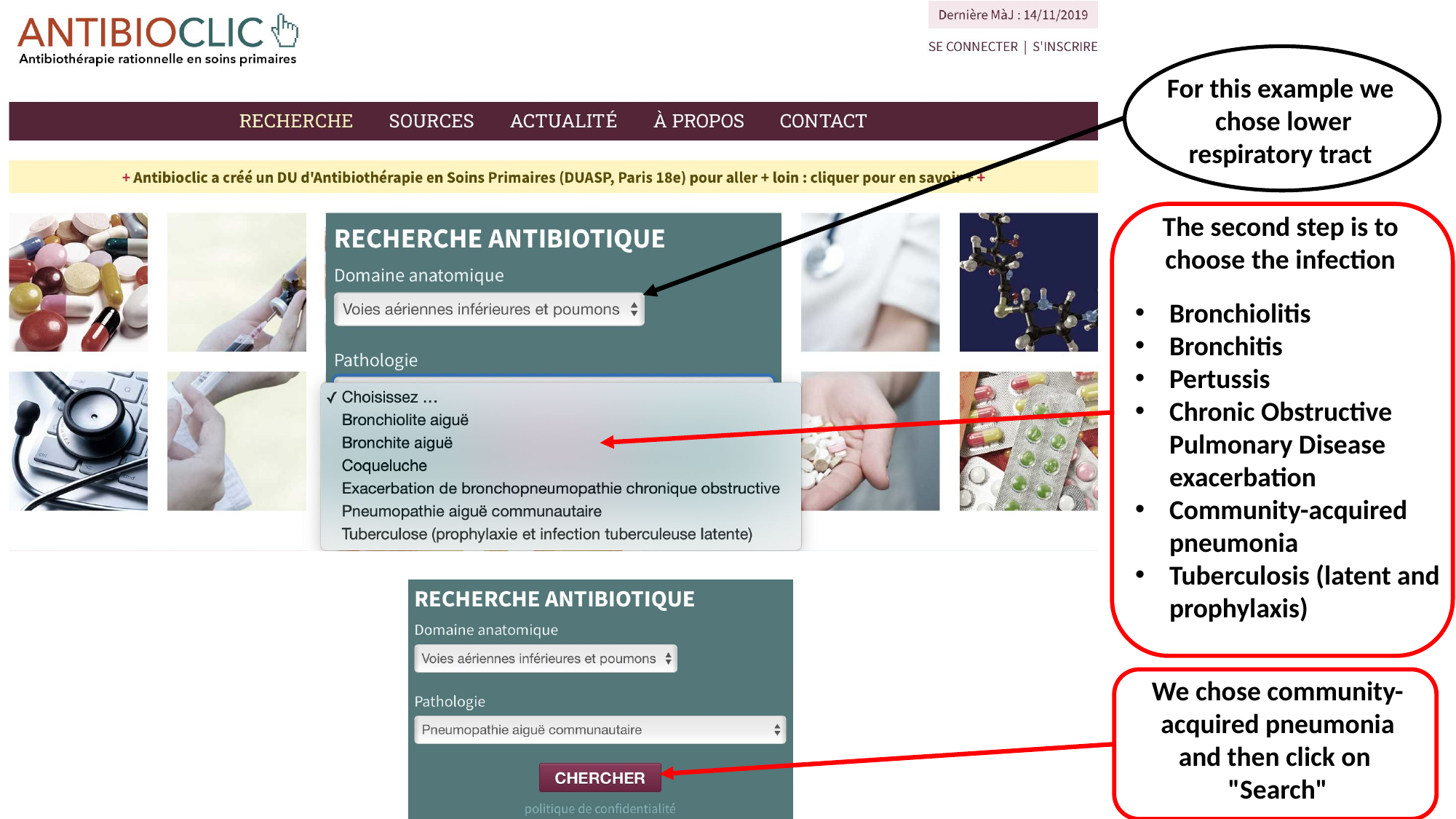

For this example we chose lower respiratory tract
The second step is to choose the infection
Bronchiolitis
Bronchitis
Pertussis
Chronic Obstructive Pulmonary Disease exacerbation
Community-acquired pneumonia
Tuberculosis (latent and prophylaxis)
We chose community-acquired pneumonia
and then click on
"Search"

## Slide 8
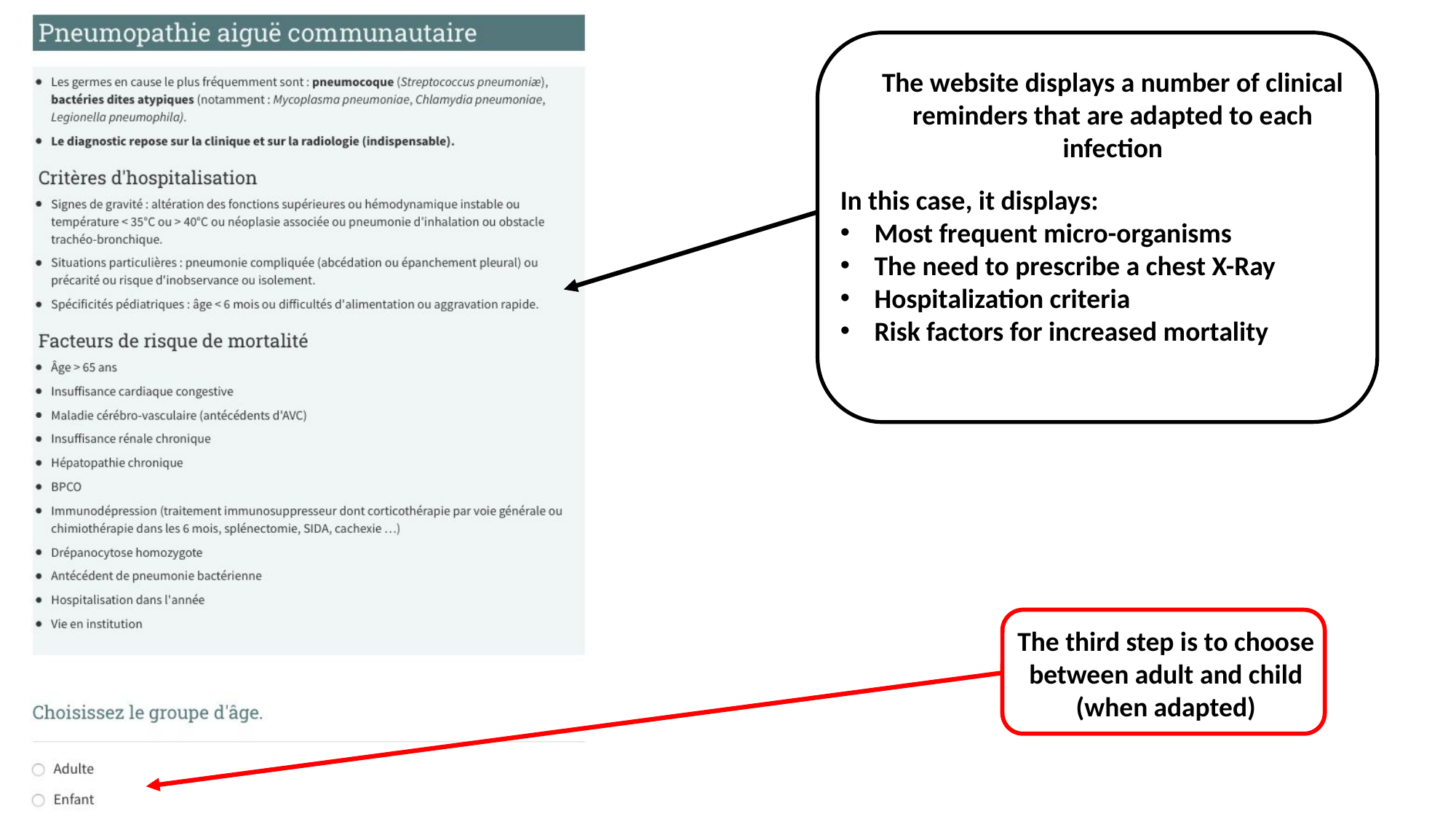

The website displays a number of clinical reminders that are adapted to each infection
In this case, it displays:
Most frequent micro-organisms
The need to prescribe a chest X-Ray
Hospitalization criteria
Risk factors for increased mortality
The third step is to choose between adult and child (when adapted)

## Slide 9
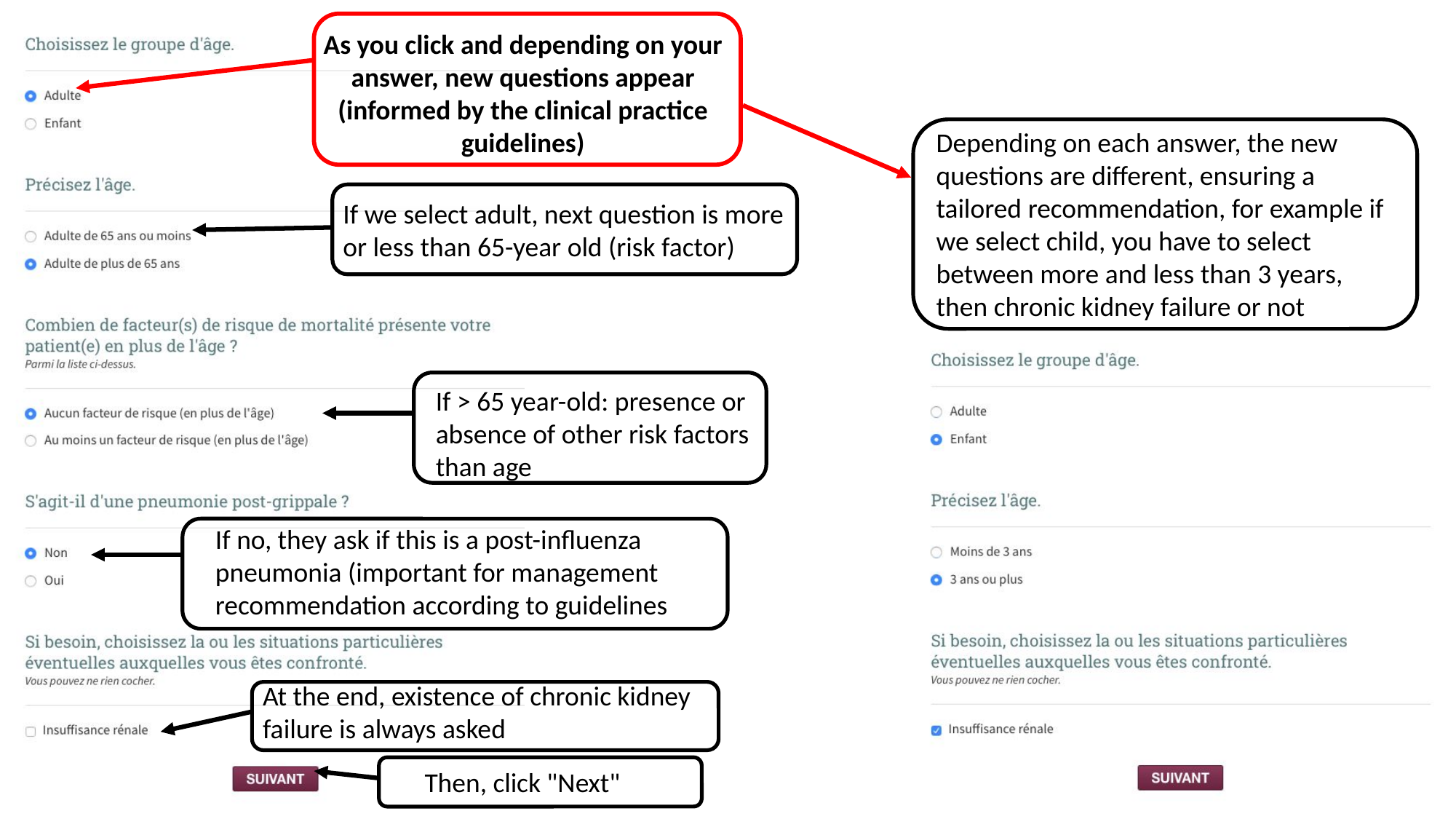

As you click and depending on your answer, new questions appear (informed by the clinical practice guidelines)
Depending on each answer, the new questions are different, ensuring a tailored recommendation, for example if we select child, you have to select between more and less than 3 years, then chronic kidney failure or not
If we select adult, next question is more or less than 65-year old (risk factor)
If > 65 year-old: presence or absence of other risk factors than age
If no, they ask if this is a post-influenza pneumonia (important for management recommendation according to guidelines
At the end, existence of chronic kidney failure is always asked
Then, click "Next"

## Slide 10
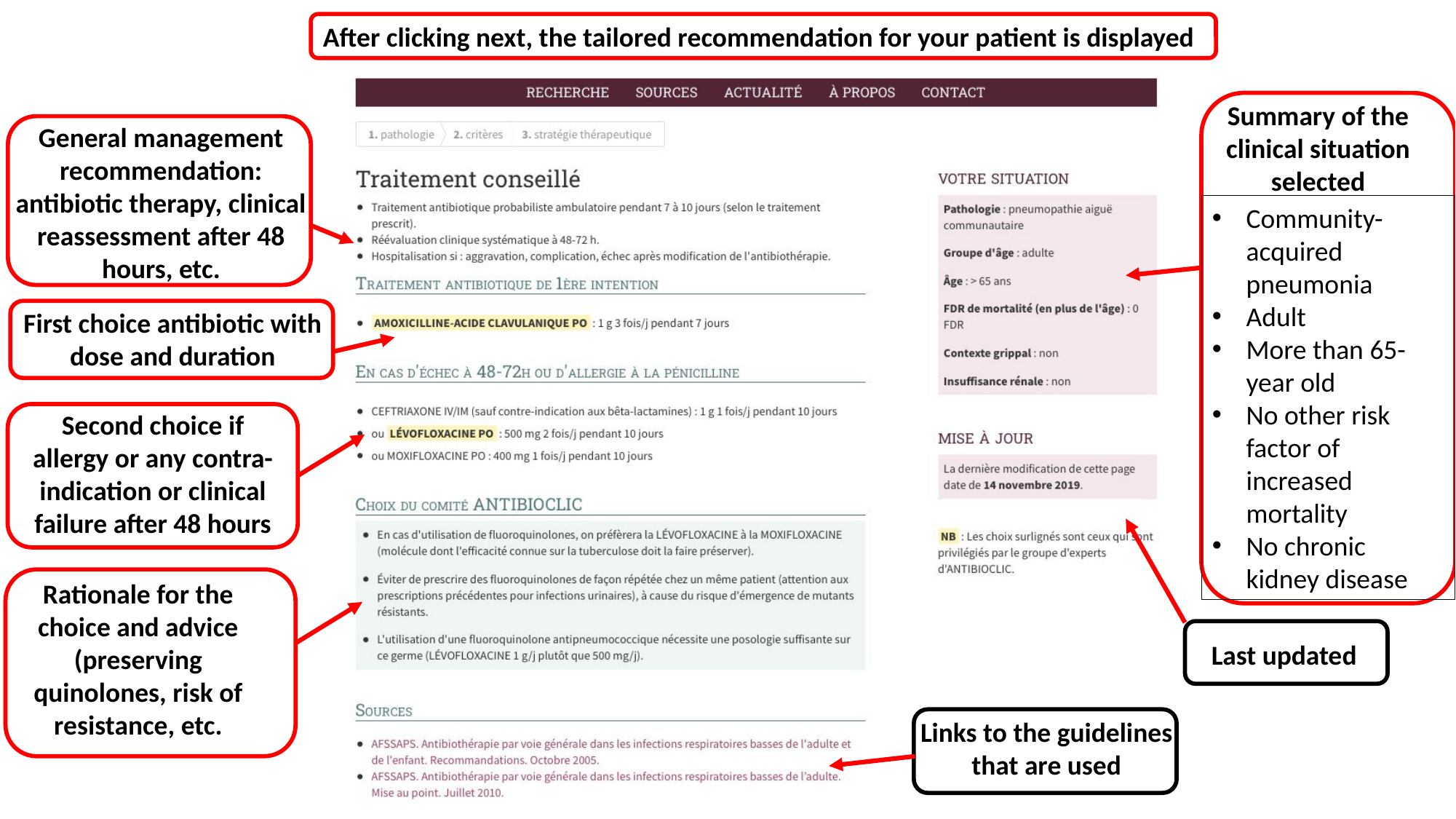

After clicking next, the tailored recommendation for your patient is displayed
Summary of the clinical situation selected
General management recommendation: antibiotic therapy, clinical reassessment after 48 hours, etc.
Community-acquired pneumonia
Adult
More than 65-year old
No other risk factor of increased mortality
No chronic kidney disease
First choice antibiotic with dose and duration
Second choice if allergy or any contra-indication or clinical failure after 48 hours
Rationale for the choice and advice (preserving quinolones, risk of resistance, etc.
Last updated
Links to the guidelines that are used

## Slide 11
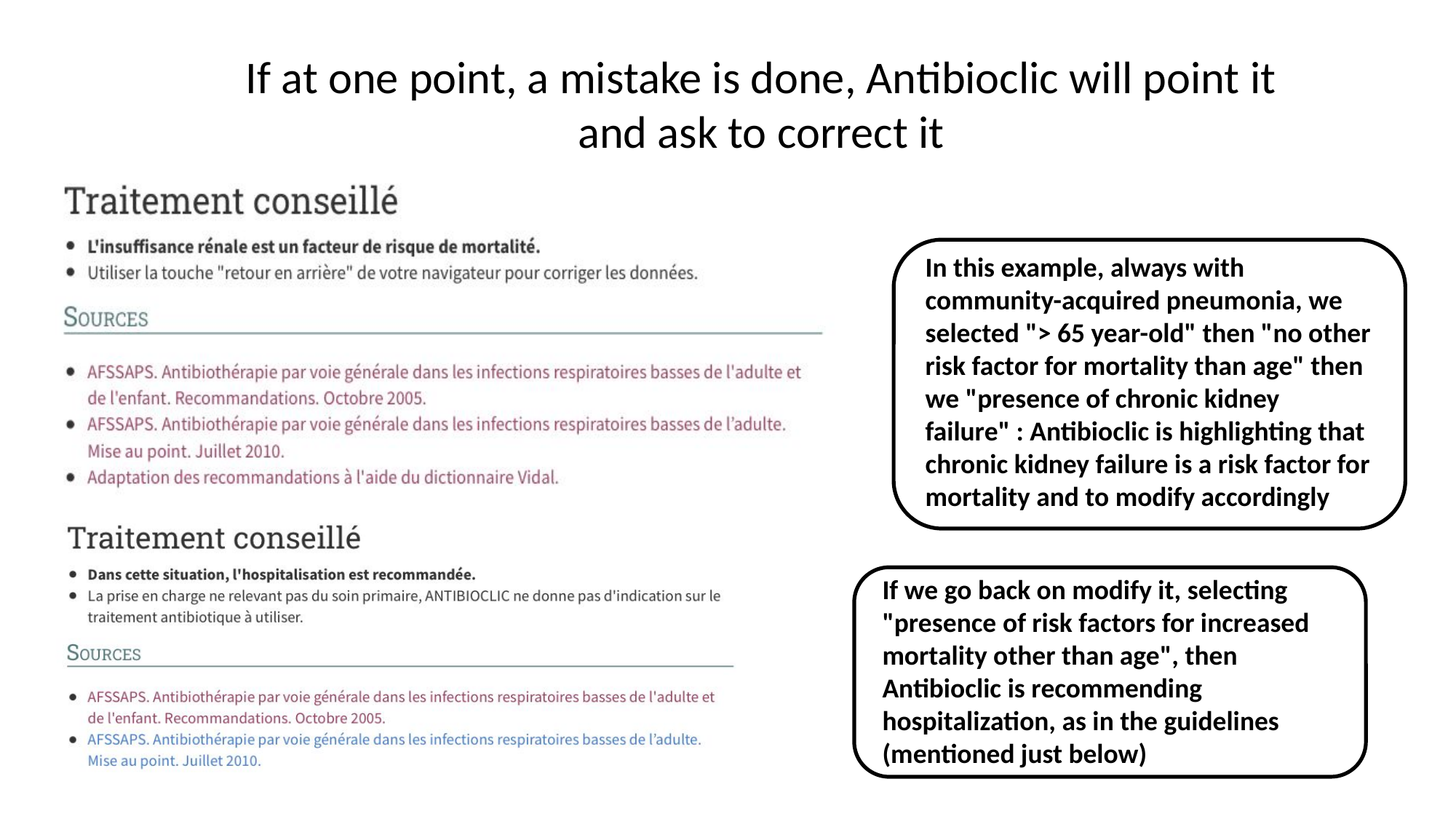

If at one point, a mistake is done, Antibioclic will point it and ask to correct it
In this example, always with community-acquired pneumonia, we selected "> 65 year-old" then "no other risk factor for mortality than age" then we "presence of chronic kidney failure" : Antibioclic is highlighting that chronic kidney failure is a risk factor for mortality and to modify accordingly
If we go back on modify it, selecting "presence of risk factors for increased mortality other than age", then Antibioclic is recommending hospitalization, as in the guidelines (mentioned just below)

## Slide 12
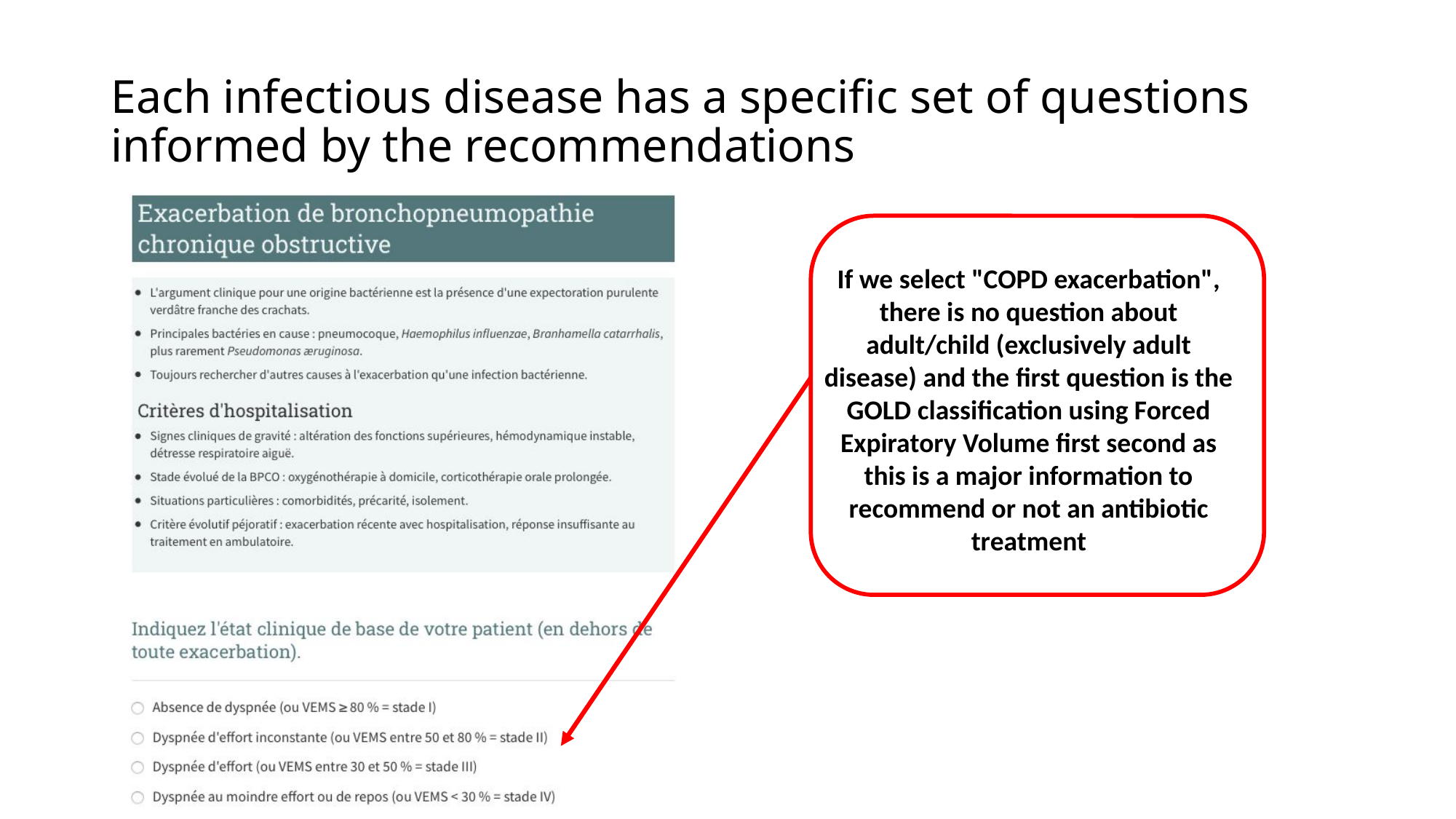

# Each infectious disease has a specific set of questions informed by the recommendations
If we select "COPD exacerbation", there is no question about adult/child (exclusively adult disease) and the first question is the GOLD classification using Forced Expiratory Volume first second as this is a major information to recommend or not an antibiotic treatment
