# Supplementary material for: Paving the Way for the Implementation of a Decision Support System for Antibiotic Prescribing in Primary Care in West Africa: Preimplementation and Co-Design Workshop With Physicians
Source: J Med Internet Res. 2020 Jul 20;22(7):e17940. doi: 10.2196/17940 (PMC7400049; doi:10.2196/17940)
Supplement: Multimedia Appendix 3 [file jmir_v22i7e17940_app3.pptx]

## Slide 1
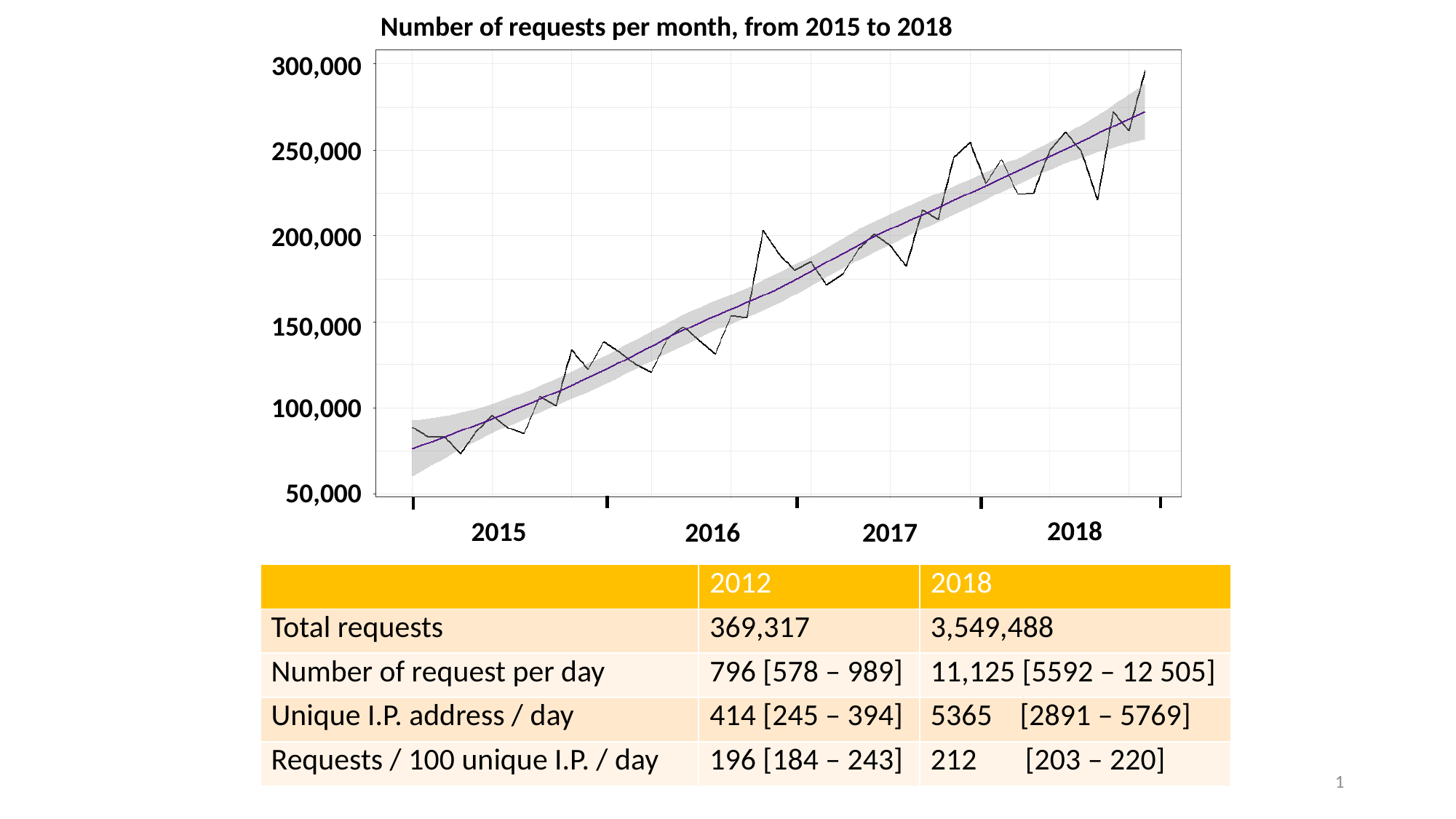

Number of requests per month, from 2015 to 2018
300,000
250,000
200,000
150,000
100,000
50,000
2018
2015
2016
2017
| | 2012 | 2018 |
| --- | --- | --- |
| Total requests | 369,317 | 3,549,488 |
| Number of request per day | 796 [578 – 989] | 11,125 [5592 – 12 505] |
| Unique I.P. address / day | 414 [245 – 394] | 5365 [2891 – 5769] |
| Requests / 100 unique I.P. / day | 196 [184 – 243] | 212 [203 – 220] |
1

## Slide 2
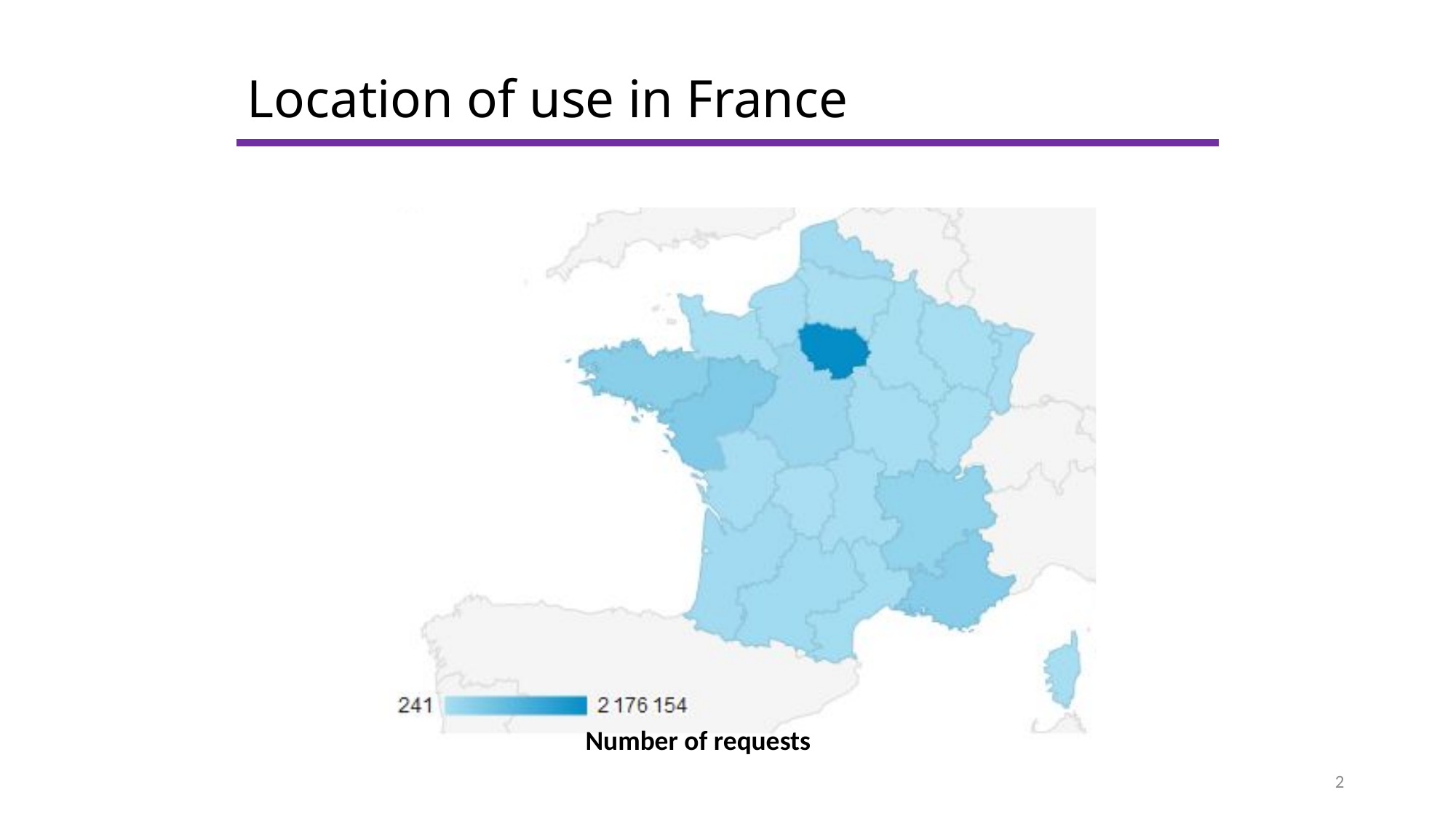

# Location of use in France
Number of requests
2
