# Supplementary material for: Paving the Way for the Implementation of a Decision Support System for Antibiotic Prescribing in Primary Care in West Africa: Preimplementation and Co-Design Workshop With Physicians
Source: J Med Internet Res. 2020 Jul 20;22(7):e17940. doi: 10.2196/17940 (PMC7400049; doi:10.2196/17940)
Supplement: Multimedia Appendix 4 [file jmir_v22i7e17940_app4.docx]

Questionnaire

You are being invited to complete this questionnaire to give your opinion on decision support systems for antimicrobial prescribing.

Please tick each box if you agree.

| 1. I confirm that I have read and understood the information given and have had the opportunity to ask questions |  |
| --- | --- |
| 2. I understand that my participation is voluntary and that I am free to withdraw at any time, without giving any reason, and without my legal rights being affected |  |
| 3. I agree to take part in this study |  |

Signature:

**General question**

What is your gender?

- Male
- Female
- Prefer to self-describe:

How old are you?

In which country do you work?

In which health structure do you work? (tick all that apply)

- Academic hospital
- Non-academic public hospital
- Private hospital
- Private surgery
- Dispensary
- Other:

What is medical specialty?

- Infectious diseases
- Intensive care
- Internal medicine
- Clinical microbiology
- Family medicine
- Other:

What is your status?

- Resident
- Doctor
- Associate professor
- Professor

Do you have national guidelines for antimicrobial prescribing?

- Yes
- No

If no, which guidelines do you usually use? (tick all that apply)

- European
- American
- French
- WHO
- Other:

Do you have access to the following devices for professional use during consultation? (tick all that apply)

- Computer
- Smartphone
- Tablet
- Other:

Do you use your phone for professional use during consultation?

- Yes
- No

Do you use a clinical information technology system during consultation?

- Yes
- No

Do you use any clinical decision support systems* in your practice?

*A clinical decision support system (CDSS) is an electronic tool that can provide users with assistance to take clinical decisions. Clinical decision support systems are sometimes called computed-based or computer-aided diagnosis or therapy. CDSS are electronic tools that help users in the diagnosis (e.g. suggesting list of diagnosis when you enter symptoms), in the work-up (e.g. suggesting a list of laboratory work-up for a patient) or in the therapy of a patient (e.g. suggesting drugs when you enter a diagnosis). Clinical decision support systems can be electronic guidelines, an alert system included in the electronic health record, a web-based platform or a stand-alone software.

- Yes
- No

If yes, which system(s)?

Do you use clinical decision support systems (CDSS) for infection management or antimicrobial prescribing?

- Yes
- No

If yes, which system(s)?

How did you know them?

What do you think of Antibioclic?

In your opinion, for which infectious diseases can a CDSS such as Antibioclic be useful?

|  | Useless | Neutral | Useful |
| --- | --- | --- | --- |
| Urinary tract infections |  |  |  |
| Genital tract infections |  |  |  |
| Meningitis |  |  |  |
| Dental infections |  |  |  |
| Upper respiratory tract infections |  |  |  |
| Lower respiratory tract infections |  |  |  |
| Gastro-intestinal tract infections |  |  |  |
| Skin and soft tissue infections |  |  |  |
| Surgical site infections |  |  |  |

Do you think that a CDSS such as Antibioclic could be used in your country?

- Yes
- No

In your opinion, what would be necessary to set up or modify for Antibioclic to be adapted to the practice of general medicine in your country?

What would be the benefits of using a CDSS such as Antibioclic in your country?

What would make it difficult to use a CDSS such as Antibioclic in your country?

What features or elements could facilitate the use and dissemination of a CDSS such as Antibioclic in your country?

Who are the health professionals who could use Antibioclic in your country? (Tick all that apply)

- General practitioners
- Infectious diseases specialists
- Intensivists
- Clinical microbiologists
- Other medical specialties
- Nurses
- Pharmacists
- Other:

For each of the following, can you tell us if you think this represents a barrier or facilitator for the use of Antibioclic in general practice in your country?

|  | Barrier | Neutral | Facilitator |
| --- | --- | --- | --- |
| Independence from the pharmaceutical industry |  |  |  |
| Adapted to the guidelines of the French National Society of Infectious Diseases |  |  |  |
| Written in French |  |  |  |
| Developed in France |  |  |  |
| Available on an electronic format (smartphone, computer) |  |  |  |
| Free |  |  |  |
| Co-designed by general practitioners and engineers |  |  |  |

Do you think that a CDSS such as Antibioclic will have the following consequences?

| It will: | Unlikely | Neutral | | | Likely |  |
| --- | --- | --- | --- | --- | --- | --- |
| Improve my medical knowledge |  |  | | |  |  |
| Increase the interaction with the patients |  |  | | |  |  |
| Lead to more personalized care |  |  | | |  |  |
| Lead to more comprehensive care |  |  | | |  |  |
| Strengthen the doctor-patient relationship |  |  | | |  |  |
| Decrease the number of medical errors |  |  | | |  |  |
| Lead to blind obedience to electronical tools |  |  | | |  |  |
| Improve clinical outcomes for patients |  | |  |  | | |
| Improve the quality of antimicrobial prescribing |  | |  |  | | |
| Save you time |  | |  |  | | |
| Decrease healthcare costs |  | |  |  | | |
| Help to fight against antimicrobial resistance |  | |  |  | | |
| Improve the adequacy of antimicrobial prescribing to guidelines |  | |  |  | | |
| Improve the care of patients with infectious diseases |  | |  |  | | |
| Decrease the duration of antimicrobial therapy |  | |  |  | | |
| Decrease the global volume of antimicrobial use |  | |  |  | | |

Do you have any comments on the study or regarding clinical decision support systems for antimicrobial prescribing?

Thank you for your participation
